# Enhanced cold tolerance mechanisms in *Euglena gracilis*: comparative analysis of pre-adaptation and direct low-temperature exposure

**DOI:** 10.3389/fmicb.2024.1465351

**Published:** 2024-10-17

**Authors:** Shuai Yuan, Wen Fu, Ming Du, Rao Yao, Dan Zhang, Chao Li, Zixi Chen, Jiangxin Wang

**Affiliations:** ^1^School of Life Sciences and Oceanography, Shenzhen University, Shenzhen, China; ^2^Hainan Chenhai Aquatic Co., Ltd., Sanya, China; ^3^Mechanical Engineering College, Xi’an Shiyou University, Xi’an, China

**Keywords:** *Euglena gracilis*, cold tolerance, low-temperature adaptation, pre-adaptation, photosynthetic efficiency, antioxidant system, osmotic regulation, membrane fluidity

## Abstract

**Introduction:**

Microalgae, known for their adaptability to extreme environments, are important for basic research and industrial applications. *Euglena*, unique for its lack of a cell wall, has garnered attention due to its versatility and the presence of bioactive compounds. Despite its potential, few studies have focused on *Euglena*’s cold adaptation mechanisms.

**Methods:**

This study investigates the cold adaptation mechanisms of *Euglena gracilis*, a microalga found in highly diverse environmental habitats, by comparing its growth, photosynthetic performance, and physiological and biochemical responses under two low-temperature cultivation modes: pre-adaptation to 16°C followed by exposure to 4°C (PreC) and direct exposure to 4°C (DirC).

**Results and discussion:**

In this study, the PreC group exhibited superior growth rates, higher photosynthetic efficiency, and more excellent antioxidant activity compared to the DirC group. These advantages were attributed to higher levels of protective compounds, enhanced membrane stability, and increased unsaturated fatty acid content. The PreC group’s ability to maintain higher cell vitality under cold stress conditions underscores the significance of pre-adaptation in enhancing cold tolerance. The findings from this research provide valuable insights into the mechanisms underlying cold adaptation in *E. gracilis*, emphasizing the benefits of pre-adaptation. These insights are crucial for optimizing the cultivation of algal species under cold stress conditions, which is essential for both biotechnological applications and ecological studies. This study not only advances our understanding of *Euglena*’s adaptive responses to low temperatures but also contributes to the broader field of algal research and its industrial exploitation.

## 1 Introduction

Microalgae are microscopic, photosynthetic organisms that can be either single-celled or multicellular phytoplankton, typically found in freshwater and marine systems and capable of surviving in extreme environments (Thapa, 2020). Compared to other biomass resources, microalgae require less space, grow and reproduce quickly, are easy to digest and absorb, and have high photonic conversion efficiency (Zhu et al., 2013). As a crucial component of aquatic life, the diversity of microalgae fluctuates seasonally. Most microalgae cells remain suspended in the water column, but during adverse environmental conditions or winter cooling, a majority die off while a few settle to the bottom to overwinter, serving as the “seeds” for the following year’s growth (Lampert, 1995; Hansson, 1996; Brunberg, 2002; Verspagen et al., 2004; Cirés et al., 2013; Kumar and Thomas, 2019; Yun et al., 2020; Xu et al., 2021). The “resurrection” phenomenon refers to microalgae rising from the sediment to the water surface in spring when conditions such as temperature, light, and nutrients become favorable. Comparisons between the resuscitation of algae in shallow and deep waters reveal that about 50% of shallow-water algae resuscitate by summer. In comparison, this proportion is only 8% in deep waters, indicating the significant influence of light on algal resuscitation (Chen et al., 2016; Guo et al., 2017; Yao et al., 2022). Therefore, studying the growth characteristics and adaptability of microalgae across seasons aids in understanding the dynamics of aquatic ecosystems and provides a foundation for research on microalgae overwintering strategies.

Environmental stress significantly impacts photosynthetic organisms’ growth and yield, potentially causing morphological, physiological, biochemical, and molecular changes. Temperature is a key environmental driver controlling phytoplankton growth and productivity, directly affecting the metabolic kinetics of phytoplankton. The content of chemical components such as proteins, lipids, and carbohydrates in phytoplankton varies with temperature (Goldman and Carpenter, 1974; Fernández-González et al., 2022). For microalgae, temperatures outside the optimal range can alter enzyme kinetics, disrupting the control of metabolic processes and energy flow and thereby affecting growth (Goldman and Mann, 1980; Pearce, 1999). Low temperatures are a critical factor limiting the growth of photosynthetic organisms, as cold stress can interfere with various physiological processes, including cell membranes, photosynthesis, respiration, and water status (Yin, 2010). Photosynthetic organisms have developed low-temperature adaptation and defense mechanisms, employing various stress-control and repair techniques. These include changes in membrane fatty acid composition, enhanced antioxidant systems, controlled photosynthesis to regulate energy balance, synthesis of chaperone proteins to refold denatured proteins, accumulation of compatible solutes to maintain cellular osmotic pressure and expression of specific gene products. The mechanisms of cold resistance in photosynthetic organisms remain a research focus (Kaur et al., 2022). Under stress, the combination of cold and light can induce significant photooxidative stress in plants and cyanobacteria, potentially causing DNA damage through denaturation (Imlay, 2013; Liu et al., 2017). Moreover, reactive oxygen species (ROS) hinder PSII repair, with both singlet oxygen and superoxide radicals being potent oxidants that can oxidize the elongation factor G involved in D1 protein production, causing oxidative damage to proteins, DNA, and lipids (Apel and Hirt, 2004; Song et al., 2014).

An adverse environment can also activate specific genes within microalgal cells, promoting the synthesis and accumulation of osmotic regulatory substances. These substances increase solute concentration and decrease water potential within cells, aiding in water absorption and ensuring continuous growth. Osmotic regulatory substances involved include proline, soluble proteins, and paramylon (Barati et al., 2019). During cold acclimation in a green alga such as *Klebsormidium flaccidum* increased cell osmotic strength can reduce dehydration induced by freezing (Nagao et al., 2008). Proline maintains an osmotic balance between cells and the environment, minimizes damage, and binds with proteins to enhance solubility and enzyme stability, protecting membrane and enzyme structures (Hosseinifard et al., 2022). By increasing proline synthesis and reducing its degradation under stress, proline accumulation allows adaptation to adverse conditions (Fedotova and Dmitrieva, 2016). Proline protects against metal-induced oxidative stress by quenching singlet oxygen and scavenging hydroxyl radicals (Barati et al., 2019).

*Euglena gracilis*, a green protist, belongs to the phylum Euglenozoa, class Euglenophyceae, and genus *Euglena*. It is a mixotrophic, unicellular eukaryote in freshwater, equipped with chloroplasts, mitochondria, and Golgi bodies. The cells of *E. gracilis* are typically spindle-shaped or slender, ranging from 20 and 100 μm in diameter. They reproduce by binary fission, lack a cell wall, and possess a specialized pellicle, providing flexibility (Leander et al., 2007; Häder et al., 2017). *E. gracilis* is highly sensitive to environmental stressors (e.g., heavy metals, high light intensity, extreme pH) and is an excellent environmental indicator (He et al., 2021). Unlike other algae, *E. gracilis* lacks a cell wall, enhancing the bioavailability of its bioactive substances, including paramylon, amino acids, unsaturated fatty acids, vitamins, and antioxidants (e.g., *β*-carotene, vitamin C). It is rich in 59 essential nutrients, promoting human growth and development, and has significant applications in health maintenance and as a high-nutrient food additive (Mokrosnop et al., 2016; Yoshida et al., 2016). Studies show that *E. gracilis* can produce bioactive substances with unique structures, offering medical benefits such as anti-tumor, antioxidant, antiviral, and immune-enhancing properties, including polyunsaturated fatty acids (PUFA), particularly eicosapentaenoic acid (EPA) and docosahexaenoic acid (DHA) (He et al., 2015). It also holds significant value in the food, aquaculture, energy, and materials sectors. With advancing research, large-scale cultivation of microalgae and the development of related bioactive metabolites are emerging as industrial directions (Mahapatra et al., 2013).

*Euglena gracilis* has vast application prospects and industrial potential, and its overwintering cells play a critical role in the subsequent year’s growth and reproduction. Thus, studying its physiological and biochemical states and metabolomics helps reveal the overwintering mechanisms. Based on the above results from studies on the temperature adaptability of microalgae, we hypothesize that *E. gracilis*, after acclimation at 16°C, can better adapt to the low temperature of 4°C. This study aims to investigate the overwintering state of *E. gracilis* under different low-temperature cultivation modes, analyze various bioactive substances, and explore the resuscitation mechanisms of overwintering cells.

## 2 Materials and methods

### 2.1 Experimental design

**Figure 1:**
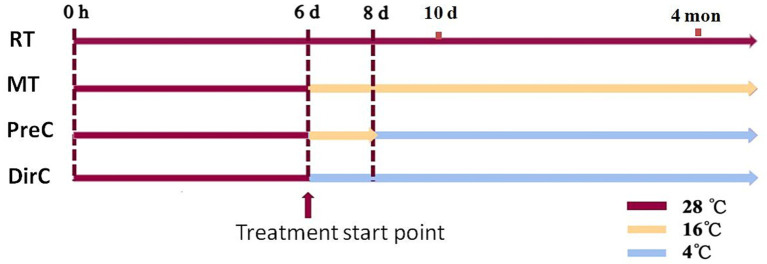
Experimental design for this study. Treatments include room temperature as control (RT), continuous 16°C (MT), 16°C followed by 4°C (PreC), and direct 4°C (DirC). 0 h, 6 d, 8 d, 10 d, 4 mon is 0 h, 6, 8, and 10 days, 4 months based on the experimental design, respectively.

*Euglena gracilis* Z strain was cultured at room temperature with light until the early stationary phase (6 d in Figure 1). Different low-temperature cultivation modes were applied: room temperature as control (RT in Figure 1), continuous 16°C (MT), 16°C followed by 4°C (PreC in Figure 1), and direct 4°C (DirC in Figure 1). Algal cells were collected on the day 8 or 10 to analyze cell morphology, growth, photosynthetic efficiency, antioxidant system (SOD activity, GSH, ascorbic acid, tocopherol content), osmotic regulation (proline, soluble protein, paramylon content), and cell membrane system (membrane composition changes and MDA content). Also, growth curves of cells from PreC and DirC treated (4 mon) culture flasks and the maximum specific growth and growth on agar plates were investigated.

### 2.2 Algal strain and culture medium

The *E. gracilis* CCAP Z strain was obtained from the College of Ocean and Life Sciences, Shenzhen University. The culture medium used was the EM autotrophic medium (Wu et al., 2021; Wu et al., 2022): 1 L medium added 1.8 g NH_4_Cl; 0.6 g KH_2_PO_4_; 0.6 g MgSO_4_; 0.06 g urea; 0.02 g CaCl_2_; 1.8 mg MnCl_2_; 1.3 mg Co(NO_3_)_2_·6H_2_O; 0.4 mg ZnSO_4_·7H_2_O; 0.2 mg H_2_MoO_4_; 0.02 mg CuSO_4_·5H_2_O; 2 mg H_2_MO_3_; 0.48 mg Na_2_EDTA; 2 mg Fe_2_(SO_4_)_3_; 0.01 mg vitamin B_1_; 0.005 mg vitamin B_12_.

### 2.3 Growth curves

To reflect the growth of *E. gracilis*, using a digital spectrophotometer (ND2000, ThermoFisher) to record the optical density (OD) at a wavelength of 750 nm. The initial content of cells was set at 10^5^ cells mL^−1^ (OD_750_ = 0.1) and measured daily until the cells reached the stationary phase.

### 2.4 Sedimentation and cell density

On day 10, 5 mL of algal suspension was gently taken from the middle of the conical flask and colorimetric tube samples for sediment experiments.

And the other cells were gently mixed and centrifuge for all culture for measuring cell density, pigment composition, and chlorophyll fluorescence efficiency. OD_750_ was measured using a microplate reader to determine cell density.

### 2.5 Pigment composition and content

1 mL of well-mixed algal suspension was centrifuged at 6000 rpm for 3 min. The supernatant was removed, and 1 mL of 95% ethanol was added. After vortexing and extraction at 4°C for 24 h in the dark, the absorbance of the extract was measured at 470, 649, and 665 nm using a UV spectrophotometer. Chlorophyll and carotenoid concentrations were calculated using standard formulas (Lichtenthaler, 1987).

$$Chl\;a\;\left(\mathrm{mg}/L\right)=13.95A665–6.88A649$$

$$Chl\;b\;\left(\mathrm{mg}/L\right)=24.96A649–7.32A665$$

$$\mathrm{Car}\;\left(\mathrm{mg}/L\right)=\left(1000A470–2.05Chl\;a-114.8Chl\;b\right)/245$$

### 2.6 Maximum chlorophyll fluorescence efficiency

15 mL of algal were kept in the dark for 30 min before measuring. The variable fluorescence (Fv) and the maximum fluorescence (Fm) were measured by pulse-amplitude modulated fluorometer (Image PAM, WALZ PHYTO-C, Germany) with a quartz cuvette. Maximum chlorophyll fluorescence efficiency was calculated as Fv/Fm = (Fm-F_0_)/Fm (Sha et al., 2019).

### 2.7 Cell morphology

On the day 10, algal suspensions were fixed with Lugol’s iodine solution and placed under an inverted microscope (DMil, Leica, Germany) to observe the morphological changes of cells with total more than 2,000 cells per treatment. The ratio of width to length was calculated and the cells were categorized into elongated shape (ratio of 0.01–0.3), spindle-shaped (ratio of 0.31–0.7), and spherical shape (ratio of 0.71–1; Kim et al., 2019).

### 2.8 Antioxidant system

Superoxide Dismutase (SOD), Glutathione (GSH), ascorbic acid (VC), and tocopherol (VE) were measured using commercial kits. The SOD assay typically utilizes a colorimetric method where the enzyme activity is determined by the inhibition of the photoreduction of nitroblue tetrazolium (NBT) in the presence of superoxide radicals generated by a riboflavin-light-NBT system. The reduction in color intensity, measured spectrophotometrically, is inversely proportional to the SOD activity in the sample. GSH levels are measured using a colorimetric assay based on the reduction of 5,5′-dithiobis(2-nitrobenzoic acid) (DTNB) by GSH to form a yellow-colored product, 5-thio-2-nitrobenzoic acid (TNB). The absorbance of TNB is measured spectrophotometrically at 412 nm, which is directly proportional to the GSH concentration in the sample. Ascorbic acid levels are typically determined using a colorimetric assay that involves the reduction of a dye such as 2,6-dichlorophenolindophenol by ascorbic acid, leading to a decrease in absorbance measured spectrophotometrically. The rate of decrease in absorbance is directly proportional to the concentration of ascorbic acid in the sample. Tocopherol is measured using a high-performance liquid chromatography (HPLC) method. The sample is first treated with an organic solvent to extract tocopherol, followed by separation on an HPLC system and detection using a fluorescence or UV detector. The peak area of tocopherol in the chromatogram is used to calculate its concentration in the sample.

### 2.9 Cell membrane system

#### 2.9.1 Total lipid content

After centrifugation, the algal cells are placed in a vacuum freeze dryer for lyophilization into powder. 100 mg of algae powder was extracted by ultrasonication for 30 min with 9.5 mL of extraction solution (chloroform: methanol: distilled water = 1:2:0.8). Adding distilled water and chloroform to the supernatant, the ratio was adjusted to chloroform: methanol: distilled water = 1:1:0.9. The organic phases were collected into a pre-weighted glass tube by centrifugation, using nitrogen gas (N2) to dry samples, and the total lipids content was obtained gravimetrically (Bligh and Dyer, 1959).

#### 2.9.2 Fatty acid content

Samples were processed for GC–MS analysis by adding extraction solvent, vortexing, and sonication. The supernatant was dried under nitrogen, and fatty acids were derivatized and re-dissolved in hexane for detection.

#### 2.9.3 MDA content

MDA was measured using a commercial kit. The basic principle of these detections involves using specific reagents that react with the target molecules to produce a colorimetric or fluorometric change, which can then be quantified spectrophotometrically to determine the concentration of the molecules in the samples.

### 2.10 Data analysis

Experiments were performed in triplicate. Data were analyzed using one-way ANOVA and Student–Newman–Keuls multiple comparison tests for significance (*p* < 0.05). Statistical analyses were performed using SPSS 16.0.

## 3 Results

### 3.1 Cell state after 4 months

#### 3.1.1 Microalgal resuscitation in liquid medium

**Figure 2:**
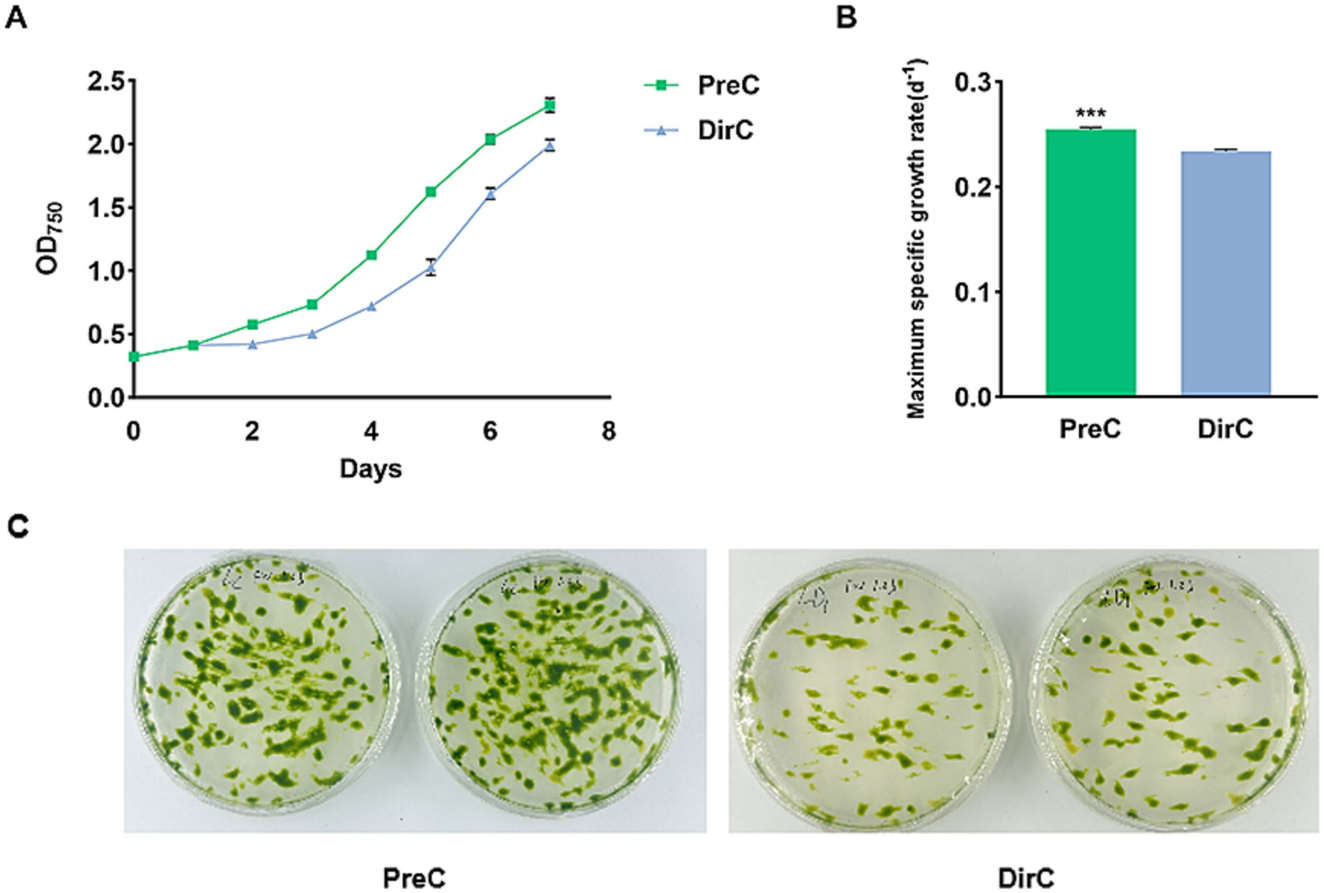
Growth curves of cells from PreC and DirC treated (4 months) (4 mon) culture flasks **(A)**, the maximum specific growth **(B)**, and growth on agar plates **(C)**.

After 4 months of simulated overwintering at 4°C, the resuscitation (transfer cells from different treatments to RT and cultivation for 7 days) growth curves of *E. gracilis* are shown in Figure 2A. It was observed that the PreC groups, pre-adapted to 16°C, had better resuscitation effects. Additionally, during the initial resuscitation period (0–2 days), all groups grew slowly, with the most significant OD value differences appearing on day 4. The PreC group maintained the cell viability of the “overwintered” *E. gracilis* cells better, resulting in faster resuscitation growth. The specific growth rate indicates the growth performance of algal cells, reflecting the vigor of various metabolic reactions within the cells. During the resuscitation period, each group’s maximum specific growth rates were measured, as shown in Figure 2B.

#### 3.1.2 Microalgae resuscitation on agar plates

Plate experiments were conducted to understand the resuscitation status of *E. gracilis* cells after low-temperature preservation. No *E. gracilis* cells were observed on the culture plates within the first 7 days, but on the 14th day, the results showed that the resuscitation under PreC was better than in DirC (Figure 2C).

### 3.2 Physiological and biochemical measurements of *Euglena gracilis*

#### 3.2.1 Cell sedimentation and photosynthetic pigments

To assess the growth vitality of *E. gracilis* at 4°C under light conditions, qualitative and quantitative measurements were performed on the PreC and DirC groups on day 10 (10 d in Figure 1). Only in the middle of the culture media was carefully pipetted up for further examination. As shown in Supplementary Figure S1, the supernatant of the PreC group cultured in conical flasks and cuvettes appeared greener than that of the DirC group, suggesting a higher cell count and better cell vitality in the supernatant (Supplementary Figure S1).

**Figure 3:**
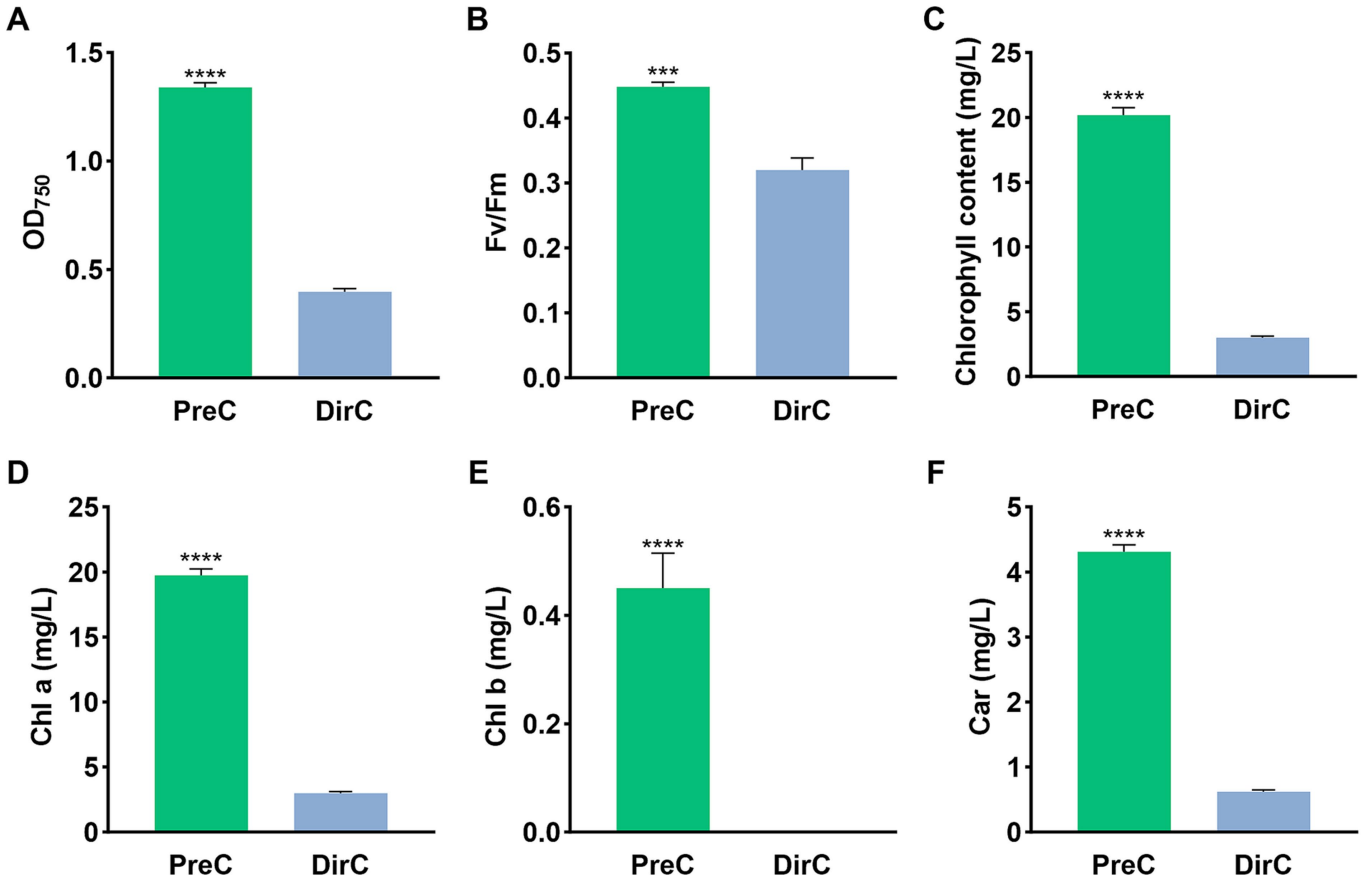
Supernatants in the middle of the flask on the day 10 were tested for OD, Fv/Fm, Chl a, and Car **(A–D)**, respectively.

In the conical flasks, the supernatant OD750 value for the PreC group was 1.34 ± 0.018, while it was 0.40 ± 0.013 for the DirC group, only 29.6% of the PreC group’s OD (Figure 3A). As shown in Figure 3C, the chlorophyll a content in the supernatant of the DirC group was significantly (*p* < 0.0001) lower than that of the PreC group, with only 30.9% of the PreC group’s content, and the carotenoid content and chlorophyll fluorescence efficiency in the supernatant of the DirC group were also significantly (*p* < 0.0001) lower than those of the PreC group.

#### 3.2.2 Cell morphology

**Figure 4:**
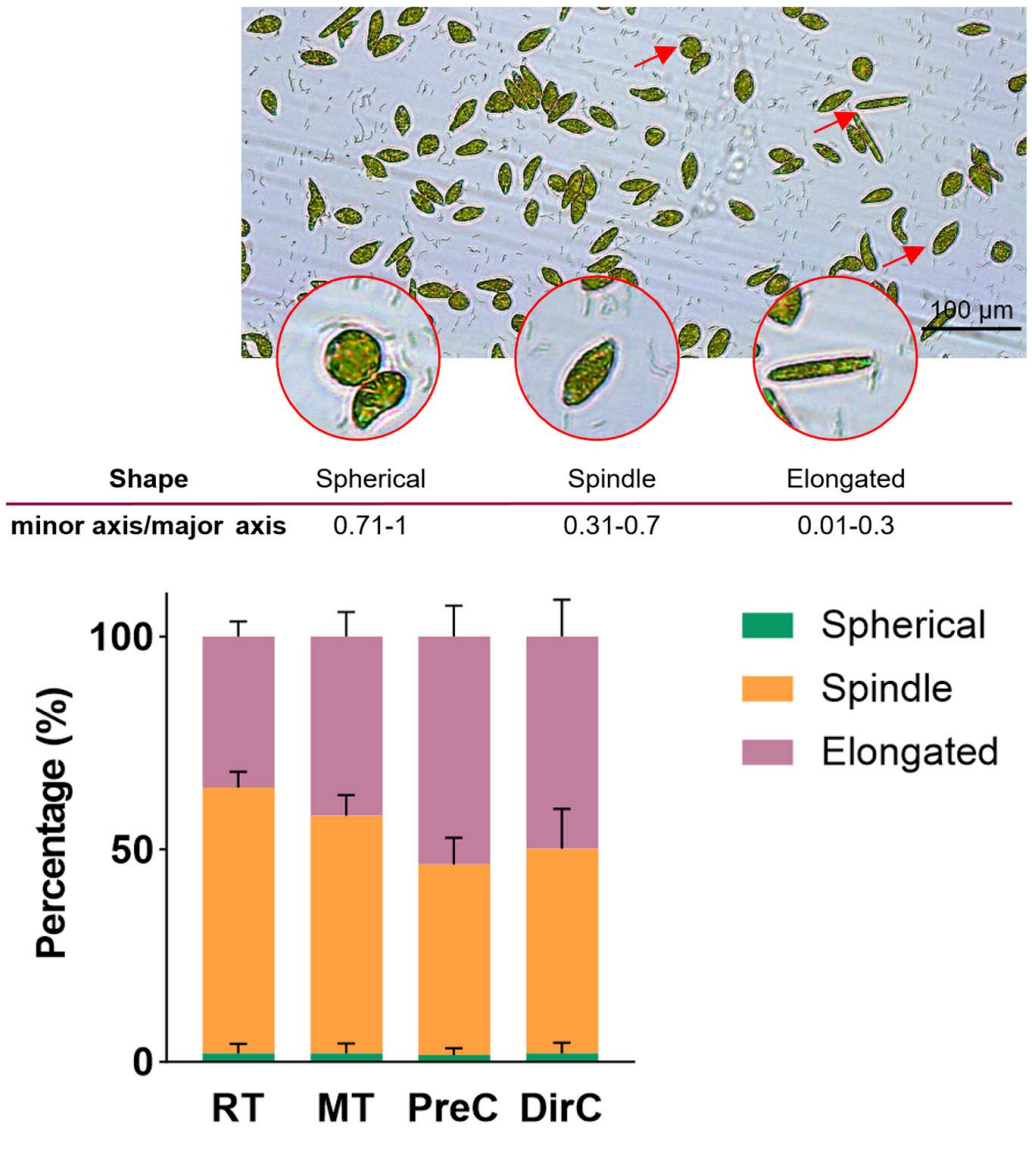
A-microscopic photos for cell sizes and shapes, B-Percentages of different cell shapes in various treatments on the day 8.

To understand the morphological changes and distribution of *E. gracilis* cells under different low-temperature culture modes, cell shapes were measured on day 10. Figure 4A shows that the proportion of spherical cells was less than 2% in all groups. 62.4 ± 3.8% of the cells in the RT group were spindle-shaped. In the MT group, slender cells accounted for about 42.1 ± 5.4%, and spindle-shaped cells accounted for 55.9 ± 4.7%, showing a similar trend to the RT group, where the proportion of spindle-shaped cells increased with the cultivation time as some slender cells became spindle-shaped. The PreC and DirC groups had similar proportions of slender and spindle-shaped cells, ranging from 44.8 ± 9.8% to 53.6 ± 6.9%, with no significant changes over time (Figure 4B).

#### 3.2.3 Photosynthetic system measurements

**Figure 5:**
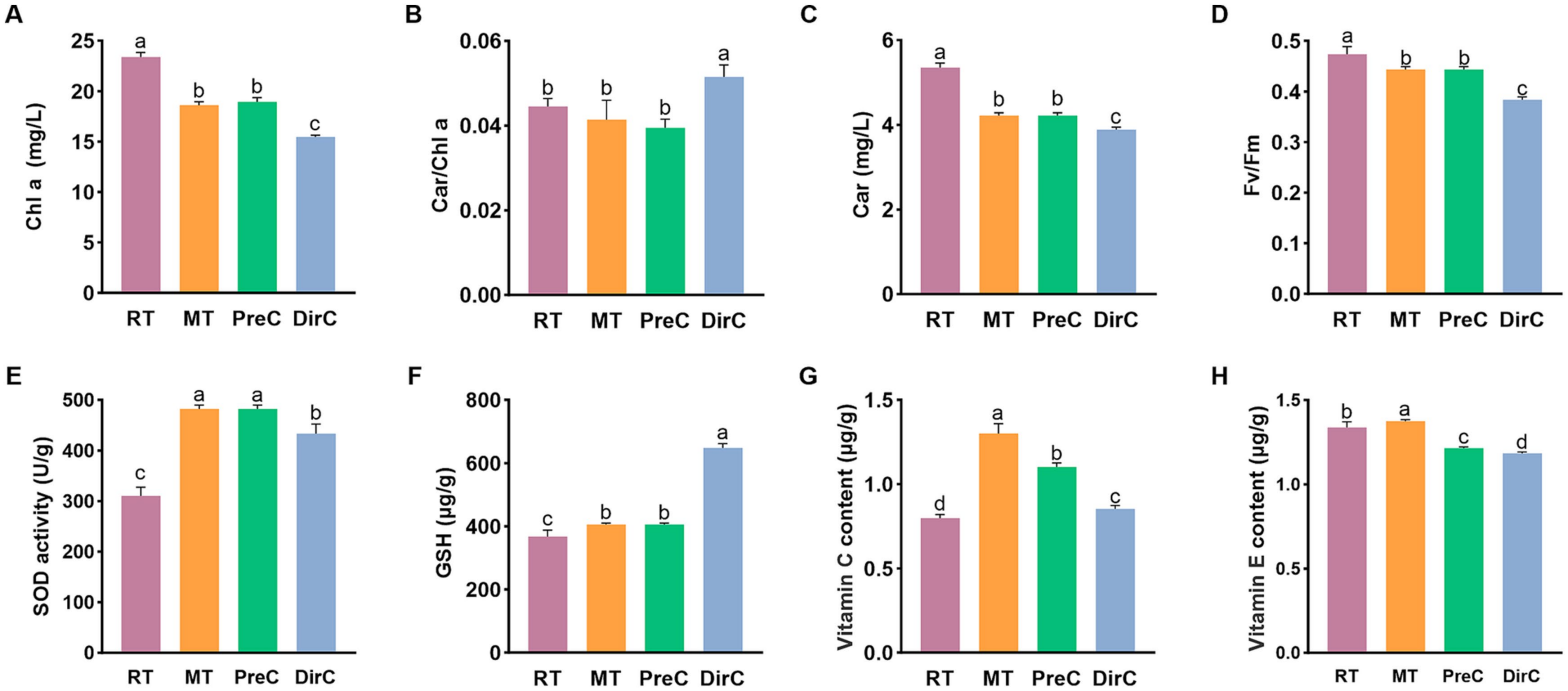
Contents of pigments (**A**-Chl a **B**-Car/Chl a, **C**-Car) and maximum photosynthetic efficiency **(D)**, antioxidants (**E**-SOD, **F**-GSH, **G**-Vitamin C, and **H**-Vitamin E) under different treatments on day 8.

The chlorophyll content reflects the photosynthetic capacity, so the photosynthetic pigment content of each group was measured. Figure 5A shows that on day 8, the control group RT had the highest chlorophyll content at 23.4 ± 0.41 mg/L. Lower cultivation temperatures significantly reduced the chlorophyll content in cells (*p* < 0.05). The MT and PreC groups, cultured at 16°C, had chlorophyll content of about 18.6–18.9 mg/L, accounting for 80.4% of the control group RT, while the DirC group, cultured directly at 4°C, had a content of 15.5 ± 0.16 mg/L, only 66.1% of the control group.

Carotenoids participate in the photosynthesis and photoprotection of oxygenic phototrophic organisms. Figure 5C shows that on day 8, the control group had the highest Car content at 5.4 ± 0.1 mg/L. The MT group, cultured at 16°C, and the DirC group, cultured at 4°C, had Car contents of about 4.2 mg/L and 3.9 mg/L, accounting for 78.9 and 72.6% of the control group, respectively. As cultivation time increased, the Car content in cells increased to some extent, while lower cultivation temperatures significantly reduced the Car content in cells (*p* < 0.05).

The Car/Chl ratio is positively correlated with the degree of photoinhibition in algal cells. As shown in Figure 5B, on day 8, the DirC group had the highest ratio, with significant differences from the other groups (*p* < 0.05).

Fv/Fm represents the maximum quantum yield of PSII and reflects the potential maximum photosynthetic capacity. A reduction in this parameter indicates that photosynthesis has been impaired. As shown in Figure 5D, on day 8, the chlorophyll fluorescence efficiency in the 28°C and 16°C groups showed no significant difference, while the DirC group, directly cultured at 4°C, had significantly lower chlorophyll fluorescence efficiency than the control group (*p* < 0.05).

#### 3.2.4 Antioxidant system measurements

SOD activity, as shown in Figure 5E, was measured to assess the antioxidant capacity of the cells. On day 8, the SOD activity in the control group cultured at 28°C was 0.015 U/10^4 cells, while the SOD activity in the cells cultured at 16°C and 4°C was significantly higher than that in the control group (*p* < 0.05) approximately 0.024 U/10^4^ cells. Additionally, the SOD activity in the PreC group was significantly higher than that in the DirC group (*p* < 0.05). The antioxidant system activity in *E. gracilis* cells increased under low-temperature cultivation, and pre-adaptation to 16°C was more conducive to resisting 4°C stress.

GSH content, as shown in Figure 5F, was also measured. On day 8, the GSH content in the DirC group, directly transferred to 4°C, was about 0.032 μg/10^4^ cells. The GSH content in the cells cultured at 28°C and 16°C was significantly lower than that in the DirC group (*p* < 0.05), about 0.02 μg/10^4^ cells, accounting for 62.5% of the DirC group’s content.

Ascorbic acid (VC) content, as shown in Figure 5G, was measured. The VC content in the control group was the lowest, about 0.8 ± 0.02 mg/mL, while the MT group had the highest VC content, about 1.3 ± 0.05 mg/mL, 1.6 times that of the control group. The PreC group had the second-highest VC content, about 1.1 ± 0.02 mg/mL, 1.3 times that of the control group, while the DirC group, directly transferred to 4°C, had a VC content of about 0.8 ± 0.02 mg/mL. There were significant differences in VC content between the groups (*p* < 0.05). Comparing the PreC and DirC groups, the PreC group had about 29.1% higher VC content than the DirC group. This indicates that low-temperature cultivation significantly increases the VC content in *E. gracilis* cells, with 16°C cultivation resulting in more ascorbic acid accumulation than 4°C cultivation, leading to more robust antioxidant capacity. Additionally, pre-adaptation to 16°C before transferring to 4°C results in higher cold adaptation than direct transfer to 4°C.

Tocopherol (VE) content, as shown in Figure 5H, was measured. The VE content in the control RT and MT groups was about 1.3 ± 0.03 mg/mL and 1.4 ± 0.01 mg/mL, respectively. The MT group had a significant increase in VE content (*p* < 0.05). In contrast, the VE content in the PreC and DirC groups, pre-adapted to 16°C and directly transferred to 4°C, was significantly lower (*p* < 0.05), about 1.2 ± 0.01 mg/mL and 1.2 ± 0.01 mg/mL, respectively, 9.1 and 11.4% lower than the control group. Comparing the PreC and DirC groups, the VE content in the PreC group was significantly higher than that in the DirC group by 0.031 mg/mL. Under low-temperature cultivation, only the VE content in *E. gracilis* cells cultured at 16°C increased, while the VE content in cells cultured at 4°C increased less. Additionally, pre-adaptation to 16°C before transferring to 4°C resulted in more vital antioxidant capacity than direct transfer to 4°C.

#### 3.2.5 Osmotic regulation system measurements

**Figure 6:**
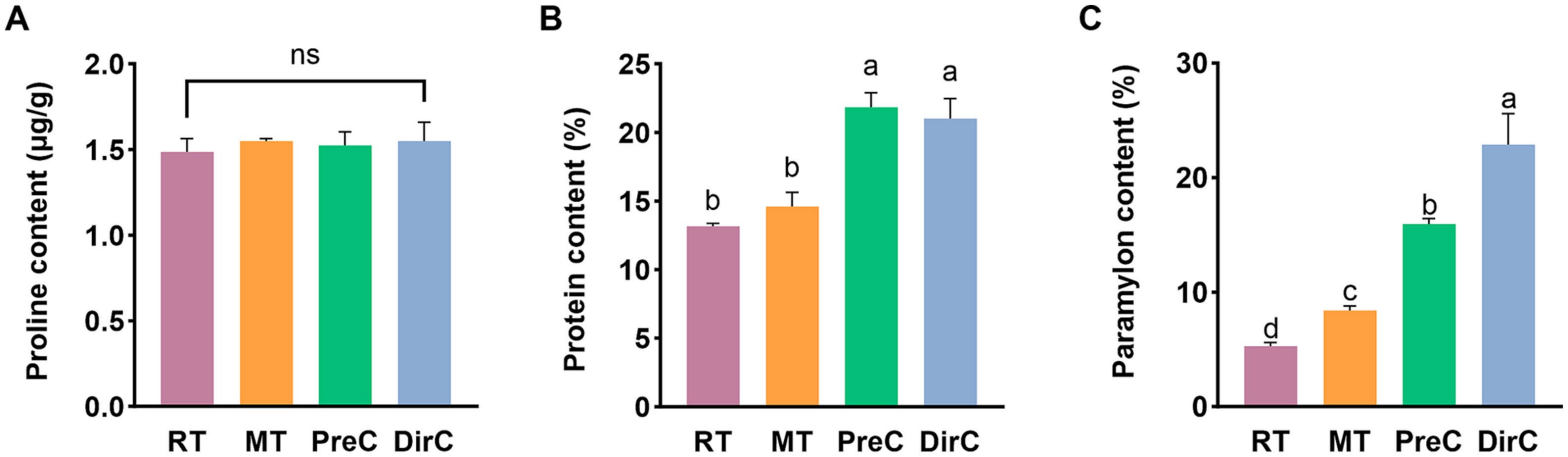
Contents of major components of osmotic system **A** proline (Pro), **B** protein content, and **C** paramylon under different treatments on day 8.

Proline is an ideal osmotic regulator. The Pro content was measured to understand the accumulation of proline in cells. As shown in Figure 6A, on day 8, there was no significant difference in Pro content between the groups transferred to low temperatures and the control group (*p* > 0.05), with Pro content of about 1.5 μg/mL in all groups. Under low-temperature cultivation, there was no significant proline accumulation in *E. gracilis* cells over a short period.

Soluble protein content reflects the extent of protein damage in plant metabolism. So as to understand the accumulation of soluble protein in *E. gracilis* cells, the soluble protein content was measured. As shown in Figure 6B, on day 8, the soluble protein content in the RT and MT groups was 13.2 ± 0.2 mg/mL and 14.6 ± 0.9 mg/mL, respectively, with no significant difference from the control group (*p* > 0.05). The soluble protein content in the PreC and DirC groups was 21.8 ± 0.9 mg/mL and 21.0 ± 1.2 mg/mL, respectively, with no significant difference between the groups (*p* > 0.05) but with a significant difference from the RT group (*p* < 0.05), 1.7 times the content of the RT group. The 16°C low temperature did not cause significant changes in the soluble protein content in cells, while 4°C cultivation significantly increased the accumulation of soluble proteins in cells.

Under adverse conditions, cells can increase the concentration of soluble sugars to enhance their resistance and withstand unfavorable environments. The content of paramylon in *E. gracilis* cells was measured. As shown in Figure 6C, on day 8, the paramylon content in the RT group was 5.3 ± 0.3%, significantly lower than that in the low-temperature groups (*p* < 0.05). The paramylon contents in the MT, PreC, and DirC groups were 8.4 ± 0.3%, 15.9 ± 0.4%, and 22.9 ± 2.2%, respectively, 1.6, 3.0, and 4.3 times that of the control group. The PreC and DirC groups had significantly higher paramylon content than the RT group (*p* < 0.05), with the DirC group having the highest paramylon content, about 1.4 times that of the PreC group.

#### 3.2.6 Cell membrane system measurements

**Figure 7:**
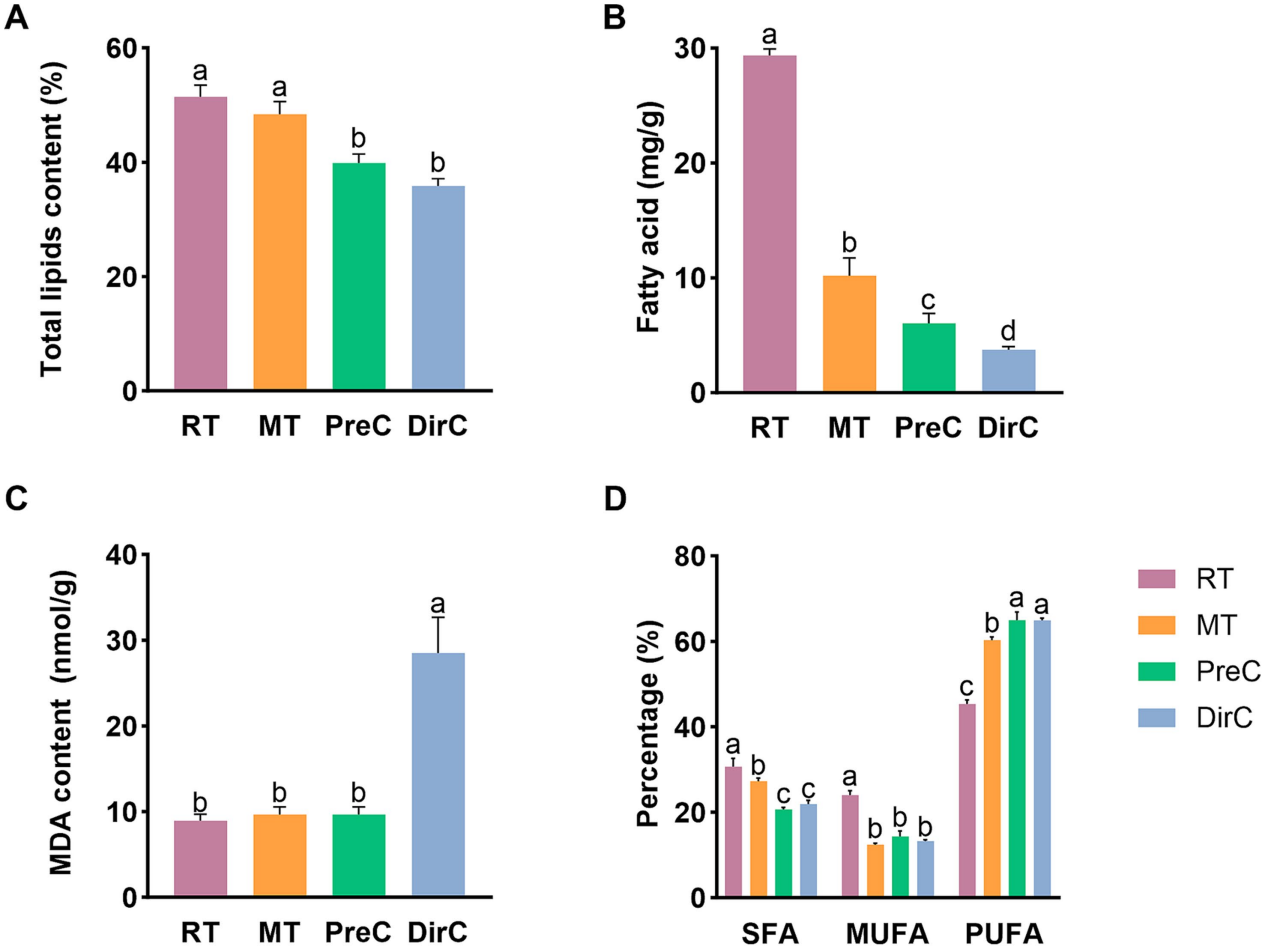
Contents of membrane-related components of **A**-total lipids, **B**-total fatty acids, **C**-MDA, **D**-SFA, MUFA, and PUFA under different treatments on day 8.

Lipids are significant metabolites in organisms and essential components of cell membranes, performing various physiological functions such as material transport, energy storage, and signal transduction. After collecting algal cells on day 8, total lipids were extracted. As shown in Figure 7A, the total lipid content in the control group was about 51.4 ± 1.7%, with no significant difference from the MT group (*p* > 0.05), about 48.4 ± 1.8%. The total lipid content in the cells cultured at 4°C in the PreC and DirC groups was significantly lower than that in the control group (*p* < 0.05), 39.9 ± 1.3% and 35.8 ± 1.0%, respectively, 77.6 and 69.7% of the control group’s content, with no significant difference between the PreC and DirC groups (*p* > 0.05). This indicates that 16°C did not cause significant changes in the total lipid content in cells, while 4°C cultivation significantly reduced the total lipid accumulation in cells. Additionally, pre-adaptation to 16°C before transferring to 4°C resulted in no significant difference in total lipid content compared to direct transfer to 4°C.

To understand the total fatty acid content in cells, total fatty acids were measured, as shown in Figure 7B. At room temperature, cells’ total fatty acid content could reach 29.3 ± 0.5 mg/g. The contents in the MT, PreC, and DirC groups were 10.2 ± 1.3 mg/g, 6.0 ± 0.7 mg/g, and 3.8 ± 0.2 mg/g, respectively, accounting for 34.7, 20.5, and 12.8% of the RT group’s content, significantly lower than that under room temperature cultivation (*p* < 0.05). Low temperatures inhibited the accumulation of total fatty acids in cells, with more significant inhibition at lower temperatures.

Malondialdehyde (MDA) is a product of membrane lipid peroxidation and an essential parameter for evaluating membrane damage and stress resistance. As shown in Figure 7C, on day 8, there was no significant difference in MDA content between the 16°C MT and PreC groups and the control group (*p* > 0.05), about 0.48 nmol/10^4^ cells. The MDA content in the DirC group, directly transferred to 4°C, was significantly higher than that in the control group (*p* < 0.05), about 1.4 nmol/10^4^ cells, 2.9 times that of the PreC group.

To understand the fatty acid content and composition under different temperature culture modes, medium and long-chain fatty acids were measured, detecting 32 fatty acids with carbon chains ranging from C12 to C22. Fatty acids were classified into saturated fatty acids (SFA), monounsaturated fatty acids (MUFA), and polyunsaturated fatty acids (PUFA) based on the presence and number of double bonds, with their content related to environmental temperature. Although the total lipid content under room temperature was significantly higher than that under low-temperature cultivation, the proportion of fatty acids varied greatly under different low-temperature conditions. The proportion of each fatty acid was shown to present a clearer picture. The PUFA proportion in the MT group was lower than in the PreC and DirC groups at 4°C. This indicates that under low-temperature cultivation, the proportion of saturated and monounsaturated fatty acids decreased, while the proportion of polyunsaturated fatty acids increased significantly.

## 4 Discussion

### 4.1 Microalgal resuscitation after 4 months’ cold treatment

Researchers have proposed the “four-stage theory” of cyanobacterial bloom formation, suggesting that cyanobacterial growth and bloom formation undergo four stages: overwintering dormancy, spring resuscitation, growth, and aggregation (Zheng et al., 2011). In this experiment, after 4 months of preservation at 4°C, resuscitation of *E. gracilis* cells was evaluated by examining growth curves, maximum specific growth rate, and photosynthetic system performance. The results showed that cells in the PreC had a more vital resuscitation ability than those in the DirC, consistent with the observed color of the supernatant in conical flasks and measured cell density. Although there was no significant difference in the photosynthetic system between the PreC and DirC groups, the PreC group outperformed the DirC group in growth curves and maximum specific growth rates. Plate experiments also showed that more cells grew in the PreC than the DirC, indicating that cells pre-adapted to 16°C before being transferred to 4°C had better cold adaptability, which aligns with other research findings. Studies have shown that plants of the same genotype exhibit a series of adaptive physiological changes after cold acclimation, significantly enhancing resistance to subsequent cold (freezing) stress (Leng and Qi, 2003). Zheng et al. (2021) found that damage to *Synechococcus* sp. PCC6803 was significantly enhanced under intense light during cold exposure. When *S.* sp. PCC6803, cultured at 30°C, was directly transferred to cold light treatment, its survival rate rapidly decreased. However, pre-cultivation at low temperature (15°C) before cold light treatment significantly improved survival rates, similar to the cold acclimation phenomenon in higher plants (Yin, 2010). When *Synechococcus* sp. PCC7942 cells were directly transferred from 37°C to 15°C for 2 h; their survival rate was less than 20%. However, if pre-treated at 25°C for 2 h before transferring to 5°C for 2 h, the survival rate increased to 80%, and even with 4 h at 15°C, the survival rate remained around 75% (Porankiewicz and Clarke, 1997).

### 4.2 Effects of different low-temperature modes on the growth of *Euglena gracilis*

In this study, under light culture conditions, the sedimentation of algal cells was compared between two different short-term 4°C low-temperature modes. Visually, the supernatant of cells pre-adapted to 16°C and then transferred to 4°C appeared greener, indicating more cells. Measurements of OD, chlorophyll composition, and maximum fluorescence efficiency in the supernatant showed that cells pre-adapted to 16°C before 4°C had better vitality and were less stressed by the low temperature. Li Jie et al. compared the physiological activity of floating and flocculated cells in Dianchi Lake and found that the electron transfer rate and NAD(P)-dependent dehydrogenase activity of flocculated cells were significantly lower than those of floating cells. Additionally, the sedimentation ratio in surface water samples reached up to 40% in winter, and the dynamic change in sedimentation ratio was negatively correlated with changes in Chl a concentration, indicating that flocculated cells were damaged and had reduced metabolic activity, similar to the results of this experiment (Li et al., 2010). The main reason for the increased sedimentation rate of microalgae is the higher proportion of sedimented cells, indirectly reflecting the deterioration of growth conditions for *Microcystis* (Jin et al., 2008). Tao et al. (2005) suggested that cell sedimentation is beneficial for the growth and reproduction of cells with high residual activity and for avoiding strong light by vertical migration. Sediment cells decompose at the bottom of the water, and the nutrients released through microbial action partially alleviate nutrient competition among surface cells. A small number of undecomposed cells enter a dormant state with reduced metabolic activity, potentially serving as the seed source for future algal blooms (Tao et al., 2005).

The shape of algal cells has been proven to be closely related to the biological clock, photosynthesis, respiration, cell cycle, and environmental factors (Lonergan, 1983). Photosynthetic and respiratory responses also participate in the daily shape changes of *E. gracilis*. The photosynthetic electron transport pathway is necessary for the shape change from spherical to slender. In contrast, the respiratory pathway is involved in both the change from spherical to slender and from elongated to spherical. The shape is also a biomarker of the cell cycle of *Euglena*, with cell length reduction possibly signaling the onset of mitosis. Additionally, the shape can change in response to various physical and chemical treatments (Li et al., 2017; Kim et al., 2019). *Euglena* exhibits several different cell morphologies, including spherical, spindle-shaped, and slender forms, representing important indicators of different cell states or physiological conditions. Most cells are spindle-shaped. In this experiment, most cells were spindle-shaped under room-temperature and low-temperature culture conditions, consistent with other research findings. At lower temperatures, some spindle-shaped cells turned into slender forms, potentially related to reduced biomass accumulation. Results indicate that treatments like *Vibrio natriegens* or Indole-3-acetic acid (IAA) affect the morphology of *E. gracilis*, especially promoting the formation of rounder cells, as stress factors force the algal cells to become round (Lonergan, 1983).

### 4.3 Effects of different low-temperature modes on the photosynthetic system of *Euglena gracilis*

When studying cold tolerance physiological indicators, chlorophyll content is commonly used as a physiological indicator to reflect the extent of low-temperature damage to plants. As the central pigment of photosynthesis, Chl a can reflect leaf photosynthetic capacity. Partelli et al. (2009) indicated that when day and night temperatures decreased from 25/20°C to 13/8°C, the Chl a content in coffee plants decreased by 30%, chlorophyll b decreased by 27%, and total chlorophyll decreased by 29%. Aghaee et al. showed that when *Oryza sativa* was exposed to low temperatures (15/10°C) for 2 weeks, total chlorophyll content decreased by 50% (Aghaee et al., 2013). In this study, the chlorophyll content in *E. gracilis* decreased by 30.7 and 42.14% under 16°C pre-adaptation, followed by 4°C cultivation and direct 4°C cultivation, respectively, consistent with the abovementioned results. The Car/Chl a ratio, which is the ratio of carotenoids to chlorophyll, is positively correlated with the degree of photoinhibition in algal cells, and the results were consistent with those of Chl a.

Carotenoids are naturally occurring pigments widely found in organisms and serve as protectors against oxidative damage by quenching free radicals and protecting chlorophyll from light damage, thereby inhibiting oxidative damage in algal cells (Barsanti et al., 2022). In this experiment, under 4°C low-temperature conditions, carotenoid content increased significantly with cultivation time, reaching about 48.9% of the control group’s content on day 10, indicating a general decrease in carotenoid accumulation compared to the control group.

Chlorophyll fluorescence efficiency (Fv/Fm) represents the maximum quantum yield of PSII photochemistry. A reduction in Fv/Fm can directly reflect the impact of stress on photosynthesis (Demetriou et al., 2007). In this experiment, chlorophyll fluorescence efficiency showed no significant change under 16°C low-temperature and room-temperature conditions. Still, it significantly decreased under 4°C low-temperature cultivation in both groups, reducing by about 26.5 and 36.3%. Pre-adaptation to 16°C resulted in higher chlorophyll fluorescence efficiency, reducing the PSII system’s light damage. Under dark conditions, chlorophyll fluorescence efficiency significantly decreased in the 4°C low-temperature group, with cells pre-adapted to 16°C showing the highest chlorophyll fluorescence efficiency. Peng et al. transferred *C. nivalis* and *C. reinhardtii* from 22°C to 4°C. They measured the maximum chlorophyll photochemical efficiency of PSII (Fv/Fm), finding that Fv/Fm in both species decreased significantly in the first 5 h. Fv/Fm in *C. nivalis* stabilized at 0.4 after 24 h, while Fv/Fm in *C. reinhardtii* dropped to 0.2 in the same period, indicating that low temperatures damaged the activity of PSII but could maintain activity at relatively low efficiency (Peng et al., 2021). Cyclic electron transport (CET) regulates the ATP/NADPH balance in photosynthetic cells and protects the photosynthetic system from high levels of light damage. At low temperatures, NDH-dependent CET is essential in alleviating oxidative damage in chloroplasts of photosynthetic organisms (Yamori et al., 2011). Although photoinhibition reduces photosynthetic electron transport efficiency, it can be seen as a protective measure for the photosynthetic apparatus in response to environmental stress, characterized by reduced Fv/Fm and dissipation of light energy as heat (Bhattacharya and Bhattacharya, 2022).

### 4.4 Effects of different low-temperature modes on the antioxidant system of *Euglena gracilis*

Environmental stress increases ROS production, highly reactive to proteins, lipids, and nucleic acids in plant cells (Mittler, 2002). Antioxidant enzymes and antioxidants can effectively scavenge intracellular ROS produced at low temperatures, maintaining cell viability. Zheng Yanli et al. found that *C. nivalis* cells are more susceptible to light stress at low temperatures. Reducing light absorption can effectively reduce light damage and enhance photoprotection to avoid ROS accumulation. They assessed the antioxidant enzyme activity and gene expression levels at 4°C, finding that SOD activity and corresponding gene expression levels in *C. nivalis* increased sharply, suggesting that antioxidant enzymes effectively scavenged intracellular ROS produced at low temperatures, maintaining cell viability (Zheng et al., 2021). In this experiment, cells cultured at 16°C had the highest overall SOD activity. SOD activity increased over a short period at 4°C, similar to other studies’ results. As cultivation time increased, the overall SOD activity of cells directly transferred to 4°C was lower than that of cells pre-adapted to 16°C and then transferred to 4°C, potentially due to lower SOD production resulting from reduced metabolism at lower temperatures. Fu Xiaoli et al. found that SOD content in unicellular *Microcystis* also increased with decreasing temperature, indicating that stress stimulated the protective capacity of the antioxidant system. Similar results were reported for *Trachydiscus minutus*, where antioxidant enzyme activity was usually inhibited at low temperatures (15°C), especially under low light (Gigova et al., 2012). Under dark and low-temperature conditions, overall SOD activity was higher than in the light group, and SOD activity in the low-temperature groups was higher than in the room temperature group, similar to results for *Scrippsiella acuminata*, where SOD activity only slightly increased. Algal cells had lower activity at low temperatures, failing to produce enough proteins and enzymes to protect against cold and dark stress (Wang et al., 2022).

GSH efficiently scavenges intracellular free radicals. In this study, GSH content in *E. gracilis* cells increased sharply under low-temperature cultivation, with GSH content in the 4°C low-temperature group approximately 62.5% of that in the room temperature group. This is similar to findings by Ding Li studying *Chlamydomonas* sp. ICE-L, where GSH content increased when *Chlamydomonas* adapted to low temperatures (8°C) after pre-adaptation to high temperatures (16°C). Studies on *Synechocystis* sp. PCC6803 have shown that GSH plays an important protective role against oxidative damage caused by environmental stress (Cameron and Pakrasi, 2010).

In 1985, Ruggeri measured tocopherol in *E. gracilis* at 12°C, finding that low-temperature stress increased *α*-tocopherol production by six to seven times, while α-tocotrienol levels decreased, with no other tocopherol homologs present (Ruggeri et al., 1985), consistent with the results of this experiment where α-tocopherol content under dark and low-temperature conditions was significantly higher than in the room temperature group. Under light 4°C low-temperature cultivation, tocopherol content was lower than in room temperature cultivation. Vismara measured tocopherol content in several microalgae in 2003, which was lower than the α-tocopherol content measured by Singh in *Porphyridium cruentum* in 2000 (Singh et al., 2000; Vismara et al., 2003). Therefore, tocopherol content in microalgae depends on temperature, light, algal species, and culture age (Hosseinifard et al., 2022). Cells pre-adapted to 16°C before transferring to 4°C had higher tocopherol content than those directly transferred to 4°C, consistent with findings by Zhang (2021) in *S.* sp. PCC6803, where pre-treatment at 15°C resulted in acquired cold-light tolerance (ACLT) stronger than direct 4°C treatment, maintaining higher tocopherol content. Gene knockout studies revealed that tocopherol is necessary for ACLT formation (Zhang, 2021). This also provides a favorable explanation for the high resuscitation activity of overwintering cells under natural winter cooling. To maintain redox homeostasis, antioxidant levels must remain within a specific range. In this experiment, ascorbic acid content significantly increased under low-temperature cultivation compared to room-temperature cultivation, enhancing antioxidant capacity. When ascorbic acid content significantly increased, the content of other antioxidants correspondingly decreased, consistent with the theory of redox homeostasis (Kanwischer et al., 2005).

### 4.5 Effects of different low-temperature modes on the osmotic regulation system of *Euglena gracilis*

Studies have shown that proline is an important cold-protective substance, with increased content enhancing cold tolerance (Goldman and Mann, 1980). In this experiment, cell proline content was insignificant under light low-temperature cultivation with 2 days of low-temperature treatment. As low-temperature cultivation time increased, proline content in cells significantly increased compared to room-temperature cultivation, possibly because proline accumulation requires time.

Paramylon, a significant energy storage substance in *E. gracilis* cells, had a content in the direct 4°C low-temperature group about 4.33 times that of the control group and 3.02 times that of the group pre-adapted to 16°C before transferring to 4°C. This indicates that lower temperatures result in higher paramylon accumulation. Increased trehalose levels have improved photosynthetic rates and reduced photooxidative damage caused by stress (Bremauntz et al., 2011). During osmotic stress, trehalose also accumulates in the *Chlorella* strain (Poong et al., 2018). Additionally, in photoautotrophic algae, starch accumulated before cell division is used as a carbon and energy reserve, ensuring the completion of the cell division process regardless of external carbon and energy supply from photosynthesis or darkness. Thus, starch degradation is an essential process in cells, especially in photosynthetic organisms during dark periods (Barati et al., 2019).

In this experiment, soluble protein content under 4°C low-temperature cultivation was significantly higher than in the room temperature group, possibly because some bacteria protect themselves from intracellular ice formation by producing antifreeze agents or ice-binding proteins, which bind to ice crystals and prevent their growth (Celik et al., 2013). Ahn et al. found that genes encoding antifreeze proteins were overexpressed at low temperatures in *Trachydiscus* (Ahn et al., 2021). Enzymes, as important components of soluble proteins, include stress-responsive and exogenous proteins. Increased soluble proteins (including antioxidant enzymes and biotransformation enzymes) may actively protect cells from low-temperature stress (Wang et al., 2022).

### 4.6 Effects of different low-temperature modes on the cell membrane system of *Euglena gracilis*

The cell membrane is a significant site of plant chilling injury, significantly affecting membrane-associated metabolic pathways such as respiratory electron transfer (Imlay, 2013). It is damaged in two ways: structural damage to protein-lipid structures, protein denaturation, and solute leakage, and adaptive changes in lipid composition under low-temperature stress are crucial for offsetting cold-induced membrane rigidity and maintaining membrane fluidity. Membrane stability also affects free electron leakage and ROS production (Sinensky, 1974; Wang and Li, 2006; Siliakus et al., 2017). Decreased temperature leads to reduced membrane fluidity, affecting membrane-associated cell functions. In some bacteria, cold induction occurs through histidine kinase cold sensors that alter membrane properties, as in *Bacillus* (Saita and De Mendoza, 2015).

Under low-temperature conditions, the composition of cell membrane lipids changes significantly, including the relative proportions of fatty acid components. These changes are closely related to membrane fluidity and stability. Microalgae lipids are classified into structural lipids, such as polyunsaturated fatty acids (PUFA), and storage lipids, such as saturated fatty acids (SFA) and monounsaturated fatty acids (MUFA). Lipids are mainly stored as triglycerides (TAG), esterified for biofuel production (Thompson, 1996). Additionally, eicosapentaenoic acid (EPA) and docosahexaenoic acid (DHA) are the most valuable fatty acids in microalgae, making them suitable for high biofuel production (Gimpel et al., 2015).

As mentioned earlier, low-temperature conditions produce ROS. Cells are equipped with antioxidant systems to mitigate the destructive impact of elevated ROS levels. However, intense environmental stress may generate ROS levels exceeding those cleared by ROS-scavenging enzymes, leading to lipid peroxidation. MDA is a product of membrane lipid peroxidation and an important parameter for evaluating membrane damage and stress resistance. Higher MDA content indicates more significant cell damage (Liu et al., 2015). In this experiment, MDA content was generally higher in low-temperature-treated groups than in the room-temperature group. Notably, MDA content rapidly increased in the PreC and DirC groups over a short period after transfer to 4°C, indicating significant stress on cells at 4°C. MDA content in cells pre-adapted to 16°C showed no significant difference from the room temperature group, possibly due to high SOD activity at 16°C reducing cell damage by clearing ROS. Studies found that MDA content in *C. reinhardtii* significantly increased at low temperatures, exhibiting very high lipid peroxidation levels, while *C. nivalis* had lower MDA content at low temperatures, with an effective ROS-scavenging mechanism, possibly an important factor in *C. nivalis* low-temperature adaptation (Zheng et al., 2021).

The physical properties of the membrane depend on its fatty acid profile and the saturation level regulating membrane fluidity, which is influenced by temperature fluctuations (Sinha and Häder, 2002). Increasing the proportion of unsaturated fatty acids at low temperatures is a well-known mechanism to maintain optimal membrane fluidity by lowering the membrane phase transition temperature (Sinensky, 1971). Besides increasing the proportion of unsaturated fatty acids, bacteria also adapt to lower temperatures by reducing chain length and increasing fatty acid branching (Ahn et al., 2021). In this experiment, low-temperature cultivation promoted a decrease in the proportion of saturated and monounsaturated fatty acids and a significant increase in the proportion of polyunsaturated fatty acids in all groups, with the proportion of polyunsaturated fatty acids increasing as temperature decreased. The proportion of unsaturated fatty acids, including cis-9-myristoleic acid, cis-9-palmitoleic acid, cis-11-octadecenoic acid, and *γ*-linolenic acid, also significantly increased. It is speculated that cells maintain membrane fluidity by increasing the unsaturation of membrane lipids. Studies have found that algae cells increase membrane unsaturated fatty acid (UFA) content at low temperatures to achieve looser lipid packing, reducing membrane lipid solidification. By increasing the percentage of unsaturated fatty acids in the membrane, the photosynthetic apparatus of algae can also adapt to cold (Barati et al., 2019). Additionally, the PreC group had higher content of 32 different fatty acids than the DirC group, suggesting that cells likely increase unsaturated fatty acid content to resist low-temperature stress. Others have found that the unsaturation of fatty acids decreases with increasing irradiance, and the percentage of total n-3 fatty acids decreased threefold, from 29 to 8% of total fatty acids, mainly due to the reduction of eicosapentaenoic acid (EPA). Temperature decrease increased the proportion of unsaturated fatty acids in algae (Mangelsdorf et al., 2009). Under 4°C light low-temperature cultivation, the proportion of polyunsaturated fatty acids significantly increased, including linoleic acid (C18:3), EPA (C20:5), DPA (C22:5), and DHA (C22:6), with EPA and DHA, which are highly nutritious, accounting for 1.598 and 4.276 times their proportions in room temperature-cultured cells. EPA and DHA content also significantly accumulated under dark conditions. This may also be a strategy for producing high-value nutritional substances EPA and DHA.

## 5 Conclusion

This study investigated the low-temperature adaptation mechanisms of *E. gracilis* through a detailed examination of cell growth, photosynthetic performance, and physiological and biochemical responses under different low-temperature cultivation modes. The key findings highlighted significant differences between the PreC and DirC groups, providing insights into the cold adaptation mechanisms of *E. gracilis*. In conclusion, pre-adaptation to moderately low temperatures significantly enhances the cold tolerance of *E. gracilis*, primarily by boosting antioxidant defenses, maintaining membrane integrity, and optimizing photosynthetic efficiency. These findings provide valuable insights into the low-temperature adaptation mechanisms of microalgae and suggest potential strategies for improving the cultivation and industrial application of *E. gracilis* under cold stress conditions.

## Data Availability

The raw data supporting the conclusions of this article will be made available by the authors, without undue reservation.
