# Supplementary figures and images for: Enhanced cold tolerance mechanisms in Euglena gracilis: comparative analysis of pre-adaptation and direct low-temperature exposure
Source: Front Microbiol. 2024 Oct 17;15:1465351. doi: 10.3389/fmicb.2024.1465351 (PMC11524907; doi:10.3389/fmicb.2024.1465351)

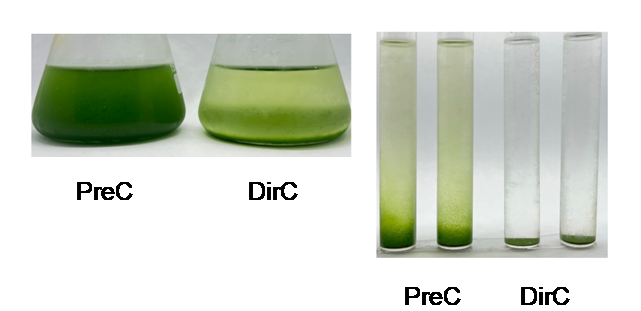

Supplement: Supplementary file 1 [file Image_1.TIF]
